# Classifying liganded states in heterogeneous single-particle cryo-EM datasets

**DOI:** 10.1093/jmicro/dfab044

**Published:** 2022-02-18

**Authors:** William R Arnold, Daniel Asarnow, Yifan Cheng

**Affiliations:** Department of Biochemistry and Biophysics, University of California San Francisco, 600 16th Street, San Francisco, CA 94158, USA; Department of Biochemistry and Biophysics, University of California San Francisco, 600 16th Street, San Francisco, CA 94158, USA; Department of Biochemistry and Biophysics, University of California San Francisco, 600 16th Street, San Francisco, CA 94158, USA; Howard Hughes Medical Institute, University of California San Francisco, 600 16th Street, San Francisco, CA 94158, USA

**Keywords:** focused classification, particle subtraction, structural heterogeneity, TRPV1, ligands, cryo-EM

## Abstract

A powerful aspect of single-particle cryogenic electron microscopy is its ability to determine high-resolution structures from samples containing heterogeneous mixtures of the same macromolecule in different conformational or compositional states. Beyond determining structures at higher resolutions, one outstanding question is if macromolecules with only subtle conformation differences, such as the same protein bound with different ligands in the same binding pocket, can be separated reliably, and if information concerning binding kinetics can be derived from the particle distributions of different conformations obtained in classification. In this study, we address these questions by assessing the classification of synthetic heterogeneous datasets of Transient Receptor Potential Vanilloid 1 generated by combining different homogeneous experimental datasets. Our results indicate that classification can isolate highly homogeneous subsets of particle for calculating high-resolution structures containing individual ligands, but with limitations.

## Introduction

As it has been demonstrated repeatedly, single-particle cryogenic electron microscopy (cryo-EM) is capable of determining multiple high-resolution structures from a heterogeneous population of molecules in mixed conformational and compositional states [1,2]. This capability relies on computational classification to sort images of individual molecules into different classes that are sufficiently homogeneous for high-resolution reconstruction [3–6]. The homogeneity and particle number in a class are two key determinants of the resolution. Extensive classification is frequently undertaken in pursuit of higher-resolution reconstructions, through isolation of increasingly rarefied subsets of homogeneous particles [4].

Classification can also be focused on a local region of the particle under study, aiming to separate subtle conformational or compositional differences [1,2,7]. We ask whether, beyond increasing nominal resolution, particles of the same protein bound with different ligands at the same ligand binding site may be reliably separated by standard 3D classification. This is challenging in practice because of the subtle differences between ligand densities and liganded protein conformations. A recent study dissecting sub-stoichiometric ligand binding [2] further promotes the derivation of ligand binding kinetics based on the ratios of particle numbers assigned to different classes. Assessing the accuracy and reproducibility of such particle number ratios, is in turn critical to understanding the accuracy of any derived kinetic or thermodynamic parameters. These questions can drive exploration of the capabilities and limitations of standard 3D classification in identifying proteins bound with distinct ligands.

Here, we use the Transient Receptor Potential Vanilloid 1 (TRPV1) ion channel as a model system to explore the potential power and limitations of computational classification in separating particles bound with different ligands in the same ligand binding site. TRPV1 is a polymodal nociceptor that can be activated by vanilloid agonists, such as capsaicin and resiniferatoxin (RTX), or inhibited by antagonists, such as capsazepine (CPZ) [8]. We have previously determined the structures of nanodisc-reconstituted TRPV1 in the resting state, bound with both double knot toxin (DkTx) and RTX, and bound with antagonist CPZ [9]. The agonist RTX and the antagonist CPZ bind TRPV1 in the same vanilloid binding pocket, which in the resting state is occupied by a phosphatidylinositol (PI) lipid. DkTx binds distal to the vanilloid pocket, at the extracellular opening of the ion permeation pore, and stabilizes the open state of the channel evoked by RTX.

In this study, we computationally combine particles from these three different datasets, followed by computational classification to separate particles into each ligand binding state. Using our prior knowledge of the origin of each particle, we quantitatively validated the accuracy of particle classification. Our results show that TRPV1 particles bound with different ligands can be separated out with high confidence. However, classification accuracy depends on subtle structural features of each ligand or protein conformation, and errors in the estimated particle distributions are too large to provide reliable quantitative information concerning ligand binding kinetics.

## Methods

### Data processing

**Fig. 1.**
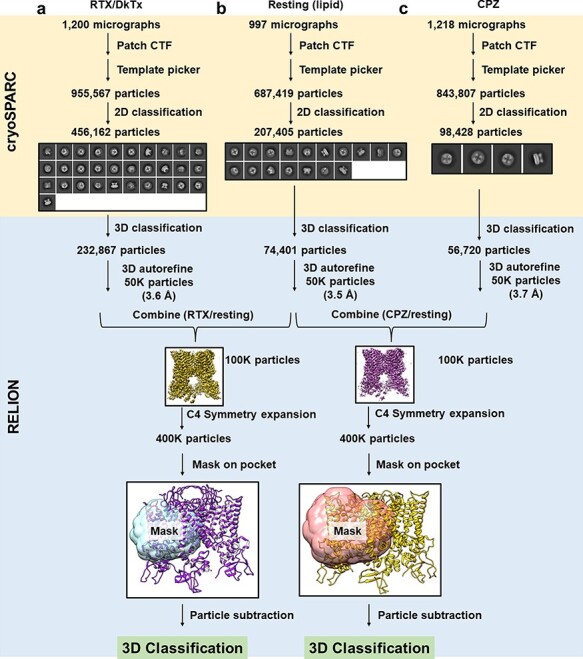
Generation of synthetic heterogeneous dataset. A flowchart showing how heterogeneous datasets were assembled by combining two individual datasets.

Using previously collected micrographs for three different samples (nanodisc-reconstituted TRPV in resting state, bound with RTX/DkTx, or CPZ [9]), we first reprocessed these datasets using now-current cryo-EM software [10–13]. We used the deposited map of resting TRPV1 in a lipid nanodisc (EMD-8118), low-pass filtered to 30 Å resolution, as the initial reference. From the final homogeneous classes of each dataset, we randomly selected 50 000 particles for further refinement and final reconstruction, yielding resolutions of 3.5 Å (resting), 3.6 Å (RTX/DkTx) and 3.7 Å (CPZ) by the 0.143 half-map Fourier Shell Correlation criterion [14]. All micrographs were collected under identical imaging conditions, and it is straightforward to combine them computationally to mimic heterogeneous datasets obtained from mixed samples. We combined particles from the DkTx/RTX and resting datasets (RTX/resting), and the CPZ and resting datasets (CPZ/resting), to generate two synthetic heterogeneous datasets that each contains 100 000 particles. 3D refinement was performed on these combined datasets to generate a consensus reconstruction from each, with nominal resolution of 3.3 Å and 3.5 Å, respectively (Supplementary Fig. S1). A visual overview of the data processing procedure is presented in Fig. 1.

**Fig. 2.**
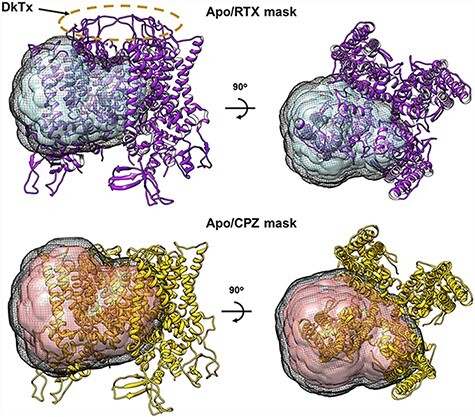
Masks used for particle subtraction and focused classification. Side (left) and top (right) views of masks overlay with ribbon diagrams of TRPV1 structures with RTX/DkTx (upper row, 5IRX) and with CPZ (lower row, 5IS0) bound. Masks cover the transmembrane domain of a monomer subunit. The black mesh shows the extent of the soft edge. The RTX/resting mask was made to exclude the DkTx by the selectivity filter. The CPZ/resting mask is the same as the RTX/resting mask but extended by 10 pixels.

The remaining data processing (classification and reconstruction) was carried out using RELION 3.1 [15]. Symmetry expansion along the C4 axis was accomplished using the relion_particle_symmetry_expand program. A mask around the vanilloid binding pocket of a TRPV1 monomer (‘monomer mask’) was made by segmenting reference density within 2 Å of the transmembrane portion of a monomer, applying a 5 Å low-pass filter and adding a soft edge of width 9.7 Å (8 pixels). The shapes of the mask for both datasets are the same, but the mask size for CPZ/resting dataset is 10 pixels (12.2 Å) larger (Fig. 2). The masks include almost the entire transmembrane domain of monomer, excluding the soluble domain and the outer pore region where DkTx binds. These masks were used for particle subtraction on the symmetry-expanded particles. The resulting symmetry-expanded/subtracted particles were then subjected to focused 3D classification without image alignments using the same monomer masks (Supplementary Fig. S2).

**Table 1.** Reconstructions of ligand and resting maps from the mixed datasets

RTX/resting	RTX sample	Resting sample	Total particles	%RTX	%Resting	Resolution
RTX class	8660	320	8980	96.4%	3.5%	3.7 Å
Resting class	525	4911	5436	9.7%	90.3%	3.9 Å
**CPZ/resting**	**CPZ sample**	**Resting sample**	**Total particles**	**%CPZ**	**%Resting**	**Resolution**
CPZ class	4052	516	4568	88.7%	11.3%	4.2 Å
Resting class	2079	6618	8697	23.9%	76.1%	4.0 Å
**RTX/CPZ**	**RTX sample**	**CPZ sample**	**Total particles**	**%RTX**	**%CPZ**	**Resolution**
RTX class	6406	205	6611	97%	3%	3.7 Å
CPZ class	268	3132	3400	8%	92%	4.2 Å

The published map of resting TRPV1 (EMD-8118) was used as the initial reference for focused classifications. We varied the degree of regularization (parameter 1/T or ‘tau fudge’ of 40 and 80) and the low-pass filter applied to the reference (12 Å or none). Classification appeared to converge after 50 iterations, and the results are summarized in Supplementary Table S1. Classes without a well-defined ligand density (PI lipid, RTX or CPZ) were marked for exclusion. The results of the symmetry-expanded particles in the remaining classes are summarized in Supplementary Table S2. To reconstruct the original tetramers from these monomeric classes, the symmetry-expanded particles from each original tetrameric particle were labeled with an identification number (0, 1, 2 or 3) and were re-grouped back to their original tetrameric particles (Supplementary Fig. S3). Tetramers that contained any number of subunits marked for exclusion were excluded. Furthermore, only particles with all four subunits being classified into same ligand classes were grouped together for calculating tetrameric reconstruction with C4 symmetry applied. The results are summarized in Table 1.

As a further validation, we generated a third synthetic dataset by combining particles from DkTx/RTX and CPZ datasets (RTX/CPZ), and performed the classification following the procedure described above, without optimizing the parameters. We used the RTX monomer mask to do the particle subtraction and classification and used a regularization parameter *T* of 40 with no low-pass filter on the reference for the classification. The results from this third synthetic dataset are included in Table 1 and Supplementary Tables S1 and S2.

### Model building

For validation purpose, we also refined the atomic models against three maps (resting, DkTx/RTX and CPZ) reprocessed in this study. The PDB (Protein Data Bank) coordinates from our previous publication (RTX/DkTx: 5IRX; Resting: 5IRZ and CPZ: 5ISO) [9] were used as the models, and incomplete prolines were fixed in ChimeraX 1.1 [16,17]. Molecular restraints were produced using the eLBOW function in Phenix 1.9.1 [18]. These models along with the restraints were then fitted into the 3D density maps via the real space refinement in Phenix using default parameters but ignoring symmetry conflicts. The models were validated for Ramachandran outliers, and none of the models were significantly different from the starting published PDB files.

## Results and discussion

**Fig. 3.**
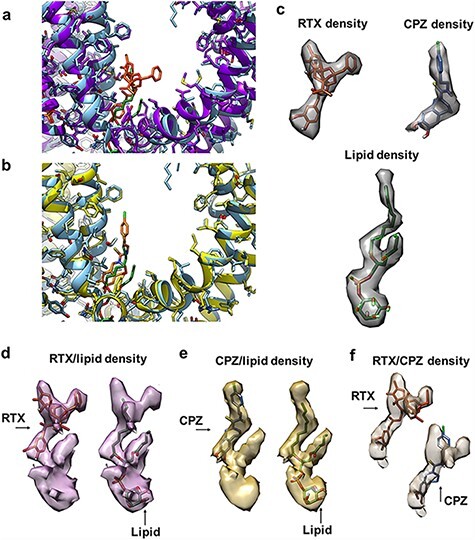
Densities of bound ligand in the vanilloid binding pocket. (a) Vanilloid binding pocket of one monomer in the resting (light blue ribbon) and RTX/DkTx (purple ribbon) states. RTX is shown as orange sticks, and the PI lipid is shown as green sticks. (b) Vanilloid binding pocket of one monomer in the resting (light blue ribbon) and CPZ (yellow) states. CPZ is shown as light orange sticks, and the PI lipid is shown as green sticks. (c) Densities for RTX, PI lipid and CPZ in the vanilloid binding pocket are well resolved in the 3D reconstructions calculated from the individual datasets. (d–f) Densities of the mixed ligands (d: RTX and PI lipid; e: CPZ and PI lipid; f: RTX and CPZ) in the vanilloid binding pocket of the 3D reconstructions calculated from the combined datasets without classification. These densities appear as the mixture of the density from individual ligand.

We have previously published the structures of nanodisc-reconstituted TRPV1 in the resting state, in which a resident PI lipid is bound in the vanilloid binding pocket; in the activated state by agonist RTX and a peptide toxin (DkTx); and the inhibited state by an antagonist CPZ [9]. In these liganded states, the resident PI lipid is displaced either by the RTX or CPZ binding to the same pocket. In contrast to a closed channel in the resting state, the DkTx/RTX bound channel is in a fully open conformation, and CPZ bound channel is in a closed conformation that is almost identical to that of the resting state (Fig. 3a and b) [9]. There is a conformational change from the resting to the fully open state, involving a subtle but noticeable near-rigid-body rotation and twist of the voltage-sensing-like domain formed by Helices S1 to S4. In addition, the density of bound DkTx in the top of the tetrameric channel constitutes another major difference among these datasets. However, this region of the structure was excluded from analysis by masking and subtraction.

While these datasets were collected several years ago, their quality is adequate for the purpose of this study. The resolutions of reconstructions determined from 50 000 particles randomly selected from the final particle stack of each dataset are comparable with each other, 3.5 Å, 3.6 Å and 3.7 Å for resting, RTX and CPZ, respectively (Fig. S1). Density within the vanilloid binding pocket of each 3D reconstruction shows a distinct characteristic shape that matches well with the known ligand, i.e. PI lipid, RTX and CPZ (Fig. 3c). The PI density is clearly defined for the phospholipid headgroup, the shoulders and the two acyl chains. The first acyl chain has well-resolved density up to about C9 of the tail; the second acyl chain density resolves up to about C4. RTX is marked by a vanilloid warhead density that overlaps with the first acyl chain of the phospholipid, followed by clear density for the phorbol ring and connected benzyl group. The CPZ density is rather similar to the first tail of the PI lipid, although the multi-ring system is wider than a bare acyl chain.

Combining the particle stacks from two different samples, we first generated two synthetic heterogeneous datasets that each contains equal number of particles from two different samples: RTX/DkTx with the resting and CPZ with the resting. Without separating the heterogeneous particles, 3D reconstruction determined from each heterogeneous dataset (Fig. S1) shows that density within the vanilloid pocket has mixed structural features of the two different ligands (Fig. 3d and e). Note that the density from CPZ/resting mixture has a shape that resembles the PI lipid, because the CPZ density overlaps almost completely with the first acyl chain of the PI lipid.

Using these two combined heterogeneous datasets, we tested a procedure that combines symmetry expansion and focused classification to separate particles of different ligand states. By comparing the classification results with the prior knowledge of which particles originated from which dataset, we quantitatively evaluate the feasibility and limitation of the classification procedure we used. The details of the procedure are described in Methods, and a conceptual description is shown in Fig. 1.

A key step in this analysis is symmetry expansion [1], which is critical to derive information concerning sub-stoichiometric ligand binding. Since the individual liganded samples were prepared separately, we can assume that the resting sample does not contain any particles bound with ligand, but the liganded sample may contain particles with sub-stoichiometric binding. To simplify analysis, we do not pursue sub-stoichiometric ligand binding in the synthetic datasets. After symmetry expansion, each tetrameric TRPV1 particle is converted to four sub-particles by assigning each particle four symmetrically equivalent alignment parameters. This is followed by applying a mask that is sufficiently large to cover the entire vanilloid binding pocket of a single monomer but small enough to exclude other particle features, particularly the density of bound DkTx, to avoid potential bias of the classification results by these distinct densities outside of vanilloid binding pocket (Fig. 2). In the procedure presented here, we also subtracted the volume that is outside of the mask. Since the remaining volume is relatively small, the classification was performed without image alignment in RELION.

We tested the mask, regularization parameter *T*, and low-pass filtering of the reference map (EMD-8118) on the classification of the symmetry-expanded particles. All classes are shown in Supplementary Fig. S2. By evaluating the appearance of ligand density in each class, we select classes with clear densities resembling the known shape of a well-resolved ligand or lipid. We then trace each particle to its sample of origin and determine the percentage of the particles that are correctly or incorrectly classified, e.g. the proportion of particles in a class with RTX features that originate in the RTX dataset or in the resting dataset (Supplementary Tables S1 and S2).

Once we determined the best conditions for classification (resulting in the greatest clarity of ligand densities), we regrouped the symmetry expanded particles back to tetrameric particles (see Supplementary Fig. S3 for a graphical outline of the procedure). For simplicity, we selected only tetrameric particles with all four subunits containing the same ligand density in the vanilloid binding pocket to calculate 3D reconstructions with C4 symmetry applied. The accuracy of separation is then estimated from the percentage of correct/incorrect particles in the final classes.

**Fig. 4.**
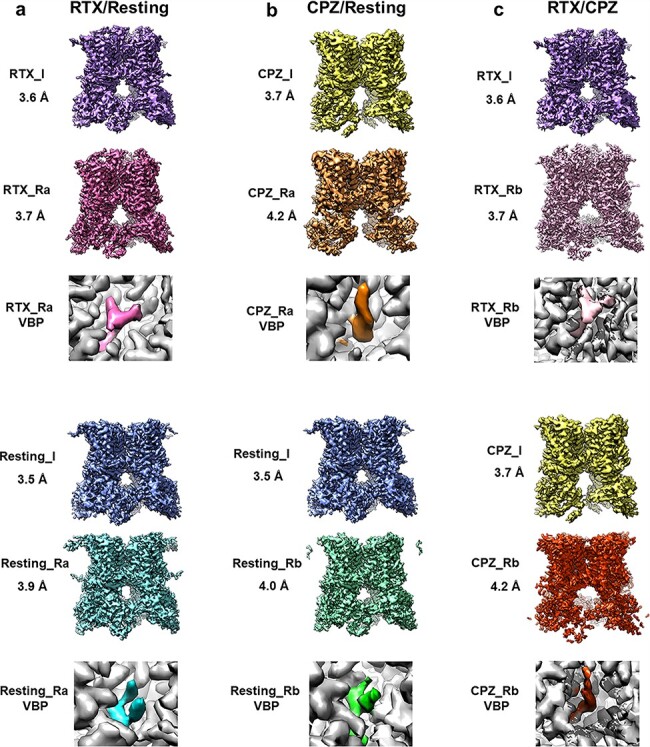
Refined tetramers after focused classification. Refinements of the homotetramers after the focused classification (_R) are compared to their respective refinements before focused classification (_I). Insets show the vanilloid binding pocket densities for the ligands (colored) and the surround protein (gray). Maps obtained after the focused classification (_R) contain similar features and similar resolutions to the maps before focused classification (_I).

For RTX/resting combined heterogeneous dataset, we were able to identify two homogeneous subsets of particles, from which calculated 3D reconstructions show a well-defined PI lipid or RTX in the vanilloid binding pocket (Fig. 4a). While each subset contains a small number of particles (8980 and 5436 out of 100 000 particles in the combined dataset), they produced reconstructions with resolution better than 4 Å and features that clearly resembles those obtained with the original, homogeneous datasets. More importantly, only a small fraction of tetramer particles is misclassified. In the RTX class, ∼3.5% of particles are misclassified from the resting state, whereas 9.6% of particles in the resting class originate from the DkTx/RTX sample. Notably, the latter group may not be exclusively misclassified, as some monomers from the liganded dataset might be ligand free due to sub-stoichiometric binding. Overall, these results suggest that the classification is largely reliable, in the sense of leading to unambiguous reconstructions of structures with alternate ligands. The full classification result is listed in Table 1.

For the CPZ/resting combined dataset, we were also able to isolate two homogeneous subsets of particles, with subunits containing either CPZ or PI lipid exclusively, respectively, comprising 4568 and 8697 out of 100 000 combined particles (Fig. 4b). The percentage of misclassified particles is larger than for RTX/resting, with 11.3% of particles in the CPZ class actually belonging to the resting sample and 23.9% of the resting class particles belonging to the CPZ dataset. While the resting and CPZ-bound structures both represent highly similar, closed-channel conformations, the higher proportion of CPZ-bound monomers misclassified as PI-bound suggests that misclassification is likely caused by the overlap of CPZ and one acyl chain of the lipid, which is difficult to distinguish at this resolution range. In contrast, the presence of the phosphoinositol headgroup may permit ready differentiation of PI from CPZ.

We also synthesized a third mixed dataset combining RTX and CPZ (RTX/CPZ), classified using the same parameters determined for the CPZ/resting data. Considering the substantial overlap of the RTX and CPZ ligands, this dataset may provide a more difficult test (Fig. 3f). We used the RTX-based VBP mask for particle subtraction and classification. The classification results are similar to what we achieved using the RTX/resting datasets, with similar particle distribution and accuracy (Supplementary Fig. S2g, Supplementary Tables S1 and S2). The density of bound ligand in RTX and CPZ classes resembles the correct shapes of these ligands (Fig. 4c, Table 1).

In the combined datasets, over 80% of total particles are excluded during the classification, because of the stringent criteria used for particle selecting and exclusion. From symmetry-expanded particles, we only select particles with all four subunits being classified in well-defined ligand classes, i.e. those with unambiguous densities within the vanilloid binding pocket. We further exclude a large portion of particles whose four subunits contained mixtures of ligand classes, i.e. some monomers in ligand classes and the others in lipid classes. The accuracies of correctly assigned particles are about 95% for the tetramers and about 60–70% for the monomers, demonstrating that selecting for homotetramers improves accuracy (Supplementary Table S1). Therefore, for proteins without symmetry the expected accuracy of similar classification may become less. Furthermore, the accuracy for the CPZ/resting dataset is lower than that for RTX/resting or RTX/CPZ, due to the amount of CPZ particles assigned to lipid classes (Supplementary Table S2). Lacking prior information concerning cooperativity of ligand binding in tetrameric channel precludes quantitative evaluation of the accuracy of sub-stoichiometric binding. Nonetheless, the procedure described here is capable of revealing channel conformations upon sub-stoichiometric ligand binding, as demonstrated recently [2]. Besides the prior knowledge of which particles originated in which sample, the resolution of final reconstruction calculated from each class is also a good indication of the reliability of the results. For the final homo-tetrameric subsets, although each contain a relatively small number of particles, the resolutions of their final reconstructions are close to the resolutions of the homogeneous datasets before focused classification.

In this study, we used RELION to perform focused classification and we noticed that the results are most influenced by the design of the mask and the regularization parameter *T*. The mask needs to be large enough to include all features of both ligand and the endogenous PI lipid and enough features of the protein. This is particularly true for the CPZ/resting combined dataset, as CPZ has a very similar shape as one tail of PI lipid. If the mask only covers this part of the particle, particles bound with CPZ will not be able to be separated from particles from the resting state. Therefore, the mask needed for the CPZ/resting classification was slightly larger than the mask needed for the RTX/resting classification; otherwise, using the RTX/resting mask on the CPZ/resting data does not produce satisfactory separation. However, a mask larger than necessary produces worse classification results, as it introduces larger common features between the liganded and the resting states. For the regularization parameter *T*, the results above were obtained with *T* = 40 for the CPZ/resting classification and *T* = 80 for the RTX/resting classification. Smaller *T* values produce worse results, with higher percentages of wrongly classified particles that produced indistinguishable classes. The focused classification can also be performed using other programs. Our results highlight that it is necessary to optimize the parameters so that the most accurate results can be produced.

Obviously, if ligand binding induces large conformational changes in the protein, the classification will be easier and more robust. In this study, the conformational difference of the transmembrane domain of individual monomer is very subtle (Fig. 3a and b). The added density of DkTx is excluded by the mask. To further remove the potential influence to the classification, we performed background subtraction. However, the procedure should work similarly without background subtraction. Furthermore, the open-channel conformation of the RTX dataset also likely contributes to the accuracy of the RTX separation, although these structural changes are relatively subtle and difficult to weigh against the ligand density differences. With all these precautions, we believe that the classification results presented here are mostly driven by the density in the vanilloid binding pocket. In this regards, better signal-to-noise ratio and higher resolution of particle image and larger dataset should help to improve the separation.

## Concluding remarks

We used TRPV1 as a model sample to ask the question if it is possible to separate particles in a compositional heterogeneous dataset that contains particles with and without ligand binding or having different ligands bound in the same binding site but without major conformational differences. Our results demonstrate that this is possible but with limitations. By applying symmetry expansion and focused classification around the ligand binding site, clean subsets of particles classes containing different ligands can be classified with good confidence to produce high-quality 3D reconstructions. The resolution of a final reconstruction is a good indicator for the success. Higher quality of data would enable better classification results and produce higher-resolution reconstruction. Symmetry expansion also allows the selection of particles with sub-stoichiometric ligand binding. However, because a large number of particles are excluded by classification, the information about ratios of particles bound with different ligands is unreliable. Thus, it is still challenging to obtain quantitative kinetic information about ligand binding. Our results suggest that it is likely possible to apply multiple ligands to the same protein targets and determine multiple structures from the same sample, which likely can improve the throughput to facilitate efficient structural-based drug development. In this case, the accuracy of the separation is most dependent on the overlap of the ligands and the overall resolution of the details that distinguish them. If ligands produce larger conformational variances, such as large domain movements, then the separation will likely be easier and may not depend on the ligands as much.

## Supplementary Material

dfab044_Supp
