# Supplementary material for: Classifying liganded states in heterogeneous single-particle cryo-EM datasets
Source: Microscopy (Oxf). 2022 Feb 18;71(Suppl 1):i23–9. doi: 10.1093/jmicro/dfab044 (PMC8855519; doi:10.1093/jmicro/dfab044)
Supplement: dfab044_Supp [file dfab044_supp.zip › Supplementary_Data_20211018_RebuttalEdits.docx]

Supplementary Data

**Classifying liganded states in heterogeneous single-particle cryo-EM datasets**

William R. Arnold^1^, Daniel Asarnow^1^, and Yifan Cheng^1,2*^

^1^Department of Biochemistry and Biophysics, University of California San Francisco, San Francisco, CA, USA

^2^Howard Hughes Medical Institute, University of California San Francisco, San Francisco, CA, USA

*Corresponding Author: yifan.cheng@ucsf.edu

Table of Contents

[Supplementary Figure 1.	3](#_Toc82705860)

[Supplementary Figure 2.	4](#_Toc82705862)

[Supplementary Figure 3..	5](#_Toc82705864)

[Supplementray Figure 4..	6](#_Toc82705866)

[Supplementary Table 1.	7](#_Toc82705867)

[Supplementary Table 2.](#_Toc82705867)	9

# Supplementary Figure 1. Maps of individual ligand states and the combined datasets.

# Supplementary Figure 2. Monomer 3D classes. (a-c) RTX/resting classes. (d-f) CPZ/resting classes. (g) RTX/CPZ classes. (a) Regularization Parameter T = 40, reference 12 Å low-pass filtered. (b) Regularization Parameter T = 40, reference not low-pass filtered. (c) Regularization Parameter T = 80, reference 12 Å low-pass filtered. This classification was used for reconstruction. (d) Regularization Parameter T = 40, reference 12 Å low-pass filtered. (e) Regularization Parameter T = 40, no low-pass filter of reference. This classification was used for reconstruction. (f) Regularization Parameter T = 80, reference not low-pass filtered. (g) Particles were subtracted using the RTX mask. Classification was performed using a regularization parameter T = 40, no low-pass filter.

# Supplementary Figure 3. Graphical outline of tetramer reconstruction script. In this work, only homotetrameric (i.e., 4:0 and 0:4) particles were used in further analysis.

# Supplementary Figure 4. Models of TRPV1 fitted into maps before focused classification (_I) and after (_R). All models were identical comparing _I to _R. Models for fitting were from the following PDB structures: RTX, PDB 5IRX; Resting, PDB 5IRZ; and CPZ, PDB 5IS0.

| Supplementary Table 1. | | |  | |  | |  | |  |  | | |  |  |
| --- | --- | --- | --- | --- | --- | --- | --- | --- | --- | --- | --- | --- | --- | --- |
| **RTX/Resting** | | | | | | | | | | | | |  |  |
| Regularization Parameter T = 40, 12 Å low-pass filter on reference | | | | | | | | | | | | |  |  |
| Class number | Ligand appearance | RTX | | Lipid | | Total in class | | % Total particles in class | | | % RTX | % Lipid |  |  |
| 1 | Low res | 9,566 | | 10,427 | | 19,993 | | 5% | | | 48% | 52% |  |  |
| 2 | Low res | 16,084 | | 11,129 | | 27,213 | | 7% | | | 59% | 41% |  |  |
| 3 | Low res | 11,116 | | 11,088 | | 22,204 | | 6% | | | 50% | 50% |  |  |
| 4 | Lipid | 36,155 | | 87,049 | | 123,204 | | 31% | | | 29% | 71% |  |  |
| 5 | Low res | 12,264 | | 17,505 | | 29,769 | | 7% | | | 41% | 59% |  |  |
| 6 | RTX | 90,150 | | 32,758 | | 122,908 | | 31% | | | 73% | 27% |  |  |
| 7 | Low res | 12,334 | | 15,399 | | 27,733 | | 7% | | | 44% | 56% |  |  |
| 8 | Low res | 12,331 | | 14,645 | | 26,976 | | 7% | | | 46% | 54% |  |  |
|  | | | | | | | | | | | | |  |  |
| Regularization Parameter T = 40, no low-pass filter on reference | | | | | | | | | | | | |  |  |
| 1 | Low res | 10,582 | | 13,182 | | 23,764 | | 6% | | | 45% | 55% |  |  |
| 2 | Low res | 13,852 | | 16,068 | | 29,920 | | 7% | | | 46% | 54% |  |  |
| 3 | Low res | 13,232 | | 17,990 | | 31,222 | | 8% | | | 42% | 58% |  |  |
| 4 | Lipid | 41,627 | | 91,055 | | 132,682 | | 33% | | | 31% | 69% |  |  |
| 5 | RTX | 86,494 | | 27,169 | | 113,663 | | 28% | | | 76% | 24% |  |  |
| 6 | Low res | 12,954 | | 11,187 | | 24,141 | | 6% | | | 54% | 46% |  |  |
| 7 | Low res | 10,664 | | 13,293 | | 23,957 | | 6% | | | 45% | 55% |  |  |
| 8 | Low res | 10,595 | | 10,056 | | 20,651 | | 5% | | | 51% | 49% |  |  |
|  | | | | | | | | | | | | |  |  |
| Regularization Parameter T = 80, 12 Å low-pass filter on reference *Used for reconstruction | | | | | | | | | | | | |  |  |
| 1 | RTX | 73,101 | | 25,019 | | 98,120 | | 25% | | | 75% | 25% |  |  |
| 2 | Lipid | 18,144 | | 29,467 | | 47,611 | | 12% | | | 38% | 62% |  |  |
| 3 | Low res | 14,217 | | 17,578 | | 31,795 | | 8% | | | 45% | 55% |  |  |
| 4 | Lipid | 26,847 | | 69,173 | | 96,020 | | 24% | | | 28% | 72% |  |  |
| 5 | RTX | 27,532 | | 19,711 | | 47,243 | | 12% | | | 58% | 42% |  |  |
| 6 | Low res | 16,699 | | 12,847 | | 29,546 | | 7% | | | 57% | 43% |  |  |
| 7 | Low res | 12,215 | | 13,270 | | 25,485 | | 6% | | | 48% | 52% |  |  |
| 8 | Low res | 11,245 | | 12,935 | | 24,180 | | 6% | | | 47% | 53% |  |  |
|  | | | | | | | | | | | | |  |  |
| **CPZ/Resting** | | | | | | | | | | | | |  |  |
| Regularization Parameter T = 40, 12 Å low-pass filter on reference | | | | | | | | | | | | |  |  |
| Class number | Ligand appearance | CPZ | | Lipid | | Total in class | | % Total particles in class | | | % CPZ | % Lipid |  |  |
| 1 | Ambiguous | 37,762 | | 33,441 | | 71,203 | | 18% | | | 53% | 47% |  |  |
| 2 | Lipid | 62,389 | | 75,152 | | 137,541 | | 34% | | | 45% | 55% |  |  |
| 3 | Low res | 6,868 | | 6,587 | | 13,455 | | 3% | | | 51% | 49% |  |  |
| 4 | Low res | 11,480 | | 11,834 | | 23,314 | | 6% | | | 49% | 51% |  |  |
| 5 | CPZ | 41,993 | | 24,828 | | 66,821 | | 17% | | | 63% | 37% |  |  |
| 6 | Low res | 5,891 | | 6,952 | | 12,843 | | 3% | | | 46% | 54% |  |  |
| 7 | Ambiguous | 26,483 | | 33,678 | | 60,161 | | 15% | | | 44% | 56% |  |  |
| 8 | Low res | 7,134 | | 7,528 | | 14,662 | | 4% | | | 49% | 51% |  |  |
|  | | | | | | | | | | | | |  |  |
| Regularization Parameter T = 40, no low-pass filter on reference *Used for reconstruction | | | | | | | | | | | | |  |  |
| 1 | Lipid | 17,757 | | 30,755 | | 48,512 | | 12% | | | 37% | 63% |  |  |
| 2 | Cpz | 41,699 | | 27,181 | | 68,880 | | 17% | | | 61% | 39% |  |  |
| 3 | Cpz | 41,953 | | 18,579 | | 60,532 | | 15% | | | 69% | 31% |  |  |
| 4 | Lipid | 23,752 | | 42,158 | | 65,910 | | 16% | | | 36% | 64% |  |  |
| 5 | Lipid | 17,756 | | 20,497 | | 38,253 | | 10% | | | 46% | 54% |  |  |
| 6 | Ambiguous | 21,753 | | 19,482 | | 41,235 | | 10% | | | 53% | 47% |  |  |
| 7 | Ambiguous | 17,032 | | 20,220 | | 37,252 | | 9% | | | 46% | 54% |  |  |
| 8 | Lipid | 18,298 | | 21,128 | | 39,426 | | 10% | | | 46% | 54% |  |  |
|  | | | | | | | | | | | | |  |  |
| Regularization Parameter T = 80, no low-pass filter on reference | | | | | | | | | | | | |  |  |
| 1 | Ambiguous | 21,975 | | 23,036 | | 45,011 | | 11% | | | 49% | 51% |  |  |
| 2 | Ambiguous | 22,270 | | 22,147 | | 44,417 | | 11% | | | 50% | 50% |  |  |
| 3 | Ambiguous | 20,157 | | 23,208 | | 43,365 | | 11% | | | 46% | 54% |  |  |
| 4 | Lipid | 42,215 | | 47,975 | | 90,190 | | 23% | | | 47% | 53% |  |  |
| 5 | Ambiguous | 20,575 | | 19,018 | | 39,593 | | 10% | | | 52% | 48% |  |  |
| 6 | CPZ | 36,546 | | 21,642 | | 58,188 | | 15% | | | 63% | 37% |  |  |
| 7 | Ambiguous | 18,509 | | 20,567 | | 39,076 | | 10% | | | 47% | 53% |  |  |
| 8 | Lipid | 17,753 | | 22,407 | | 40,160 | | 10% | | | 44% | 56% |  |  |
|  | | | | | | | | | | | | |  |  |
| **RTX/CPZ** | | | | | | | | | | | | |  |  |
| Regularization Parameter T = 40, no low-pass filter on reference. RTX mask. | | | | | | | | | | | | |  |  |
| Class number | Ligand appearance | CPZ | | Lipid | | Total in class | | % Total particles in class | | | % CPZ | % Lipid |  |  |
| 1 | Low res | 11607 | | 14175 | | 25782 | | 6% | | | 45% | 55% |  |  |
| 2 | Low res | 11602 | | 13453 | | 25055 | | 6% | | | 46% | 54% |  |  |
| 3 | RTX | 85805 | | 33891 | | 119696 | | 30% | | | 72% | 28% |  |  |
| 4 | Low res | 13019 | | 15962 | | 28981 | | 7% | | | 45% | 55% |  |  |
| 5 | CPZ | 38379 | | 82759 | | 121138 | | 30% | | | 32% | 68% |  |  |
| 6 | Low res | 15688 | | 12451 | | 28139 | | 7% | | | 56% | 44% |  |  |
| 7 | Low res | 12267 | | 14208 | | 26475 | | 7% | | | 46% | 54% |  |  |
| 8 | Low res | 11633 | | 13101 | | 24734 | | 6% | | | 47% | 53% |  |  |

Supplementary Table 2. Confusion matrices for monomer classes used in reconstruction. “Distinguishable” here refers to particles with clear ligand densities.

| **RTX/Resting** | | |
| --- | --- | --- |
| Reg. Parameter T = 80, low-pass filter 12 Å. | | |
| Counts | RTX Classes | Lipid Class |
| RTX sample | 100,633 | 44,991 |
| Lipid sample | 44,730 | 98,640 |
|  | | |
| % distinguishable particles | RTX Classes | Lipid Class |
| RTX sample | 34.8 | 15.6 |
| Lipid sample | 15.5 | 34.1 |
|  | | |
| % all particles | RTX Classes | Lipid Class |
| RTX sample | 25.2 | 11.2 |
| Lipid sample | 11.1 | 24.7 |
|  | | |
| **CPZ/Resting** | | |
| Reg. Parameter T = 40, no low-pass filter. | | |
| Counts | CPZ classes | Lipid classes |
| CPZ sample | 83,652 | 77,563 |
| Lipid sample | 45,760 | 114,538 |
|  |  |  |
| % distinguishable particles | CPZ classes | Lipid classes |
| CPZ sample | 26.0 | 24.1 |
| Lipid sample | 14.2 | 35.6 |
|  |  |  |
| % all particles | CPZ classes | Lipid classes |
| CPZ sample | 20.9 | 19.4 |
| Lipid sample | 11.4 | 28.6 |
|  | | |
| **RTX/CPZ** | | |
| Reg. Parameter T = 40, no low-pass filter. RTX mask. | | |
| Counts | RTX class | CPZ class |
| RTX sample | 85,805 | 38,379 |
| CPZ sample | 33,891 | 82,759 |
|  | | |
| % distinguishable particles | RTX class | CPZ class |
| RTX sample | 35.6 | 15.9 |
| CPZ sample | 14.1 | 34.1 |
|  | | |
| % all particles | RTX class | CPZ class |
| RTX sample | 21.5 | 9.59 |
| CPZ sample | 8.47 | 20.7 |
